# Editorial: Inflammation in hypertensive disorders

**DOI:** 10.3389/fphys.2022.1085856

**Published:** 2023-01-09

**Authors:** Hana A. Itani, Louise C. Evans

**Affiliations:** ^1^ 1Department of Pharmacology and Toxicology, Faculty of Medicine, American University of Beirut, Beirut, Lebanon; ^2^ Department of Surgery, University of Minnesota, Minneapolis, MN, United States

**Keywords:** hypertension, inflammation, diet, therapeuctic targets, memory T cell

## Introduction

Hypertension is a leading cause of global mortality and is the primary modifiable risk factor for renal, cardiovascular, and cerebrovascular disease (Kearney et al., 2005). Non-etheless, the factors regulating the pathogenesis and progression of hypertension remain incompletely elucidated. In recent years the pivotal role of the immune system in the development of hypertensive disorders has been increasingly recognized (Harrison et al., 2011; Mattson, 2014; Madhur et al., 2021; Ertuglu and Kirabo, 2022). Renal immune cells are a common feature of both experimental and clinical hypertension (Hughson et al., 2008; Crowley et al., 2010; De Miguel et al., 2011; Xiao et al., 2015; Evans et al., 2017; Banek et al., 2019; Maaliki et al., 2022). Lymphocytes and macrophages migrate to regions of injury in the kidney, and immunosuppression, whether pharmacologically or genetically induced, reduces blood pressure in preclinical models (Guzik et al., 2007; Boesen et al., 2010; Crowley et al., 2010; Madhur et al., 2010; Mattson et al., 2013; Moes et al., 2018). The articles by Itani et al. and Chaundhari et al. in this Research Topic focus on the involvement of the immune system in the development of hypertension.

While the factors driving hypertension are varied and complex, what is clear is that the prevalence of the condition is increasing. This increase is most notable in low and middle-income countries. It is hypothesized to be the result of a dietary shift from a Mediterranean-style diet to a western-style diet, high in sodium and fructose (Schutte et al., 2021). The role that diet plays in the development and prevalence of hypertension is the focus of the review by Labban et al.

## Memory T cells in hypertension

Memory T cells are a hallmark of the adaptive immune system. They mount a rapid immune response to recurrent antigen exposure. The migration of T cells from lymphoid organs to the circulation depends on the interaction between the chemoattractant sphingosine-1-phosphate (S1P) and sphingosine-1-phosphate receptors one and 2 (S1PR1+2). FTY720 is a functional agonist of S1PR1 that prevents the egression of lymphocytes from secondary lymphoid organs to the circulation (Matloubian et al., 2004; Garris et al., 2014; Itani et al., 2022).

The report by Itani et al. uses FTY720 to examine the role of effector memory T cells in the hypertensive response to repeated high-salt challenges. Mice were treated with l-NAME to inhibit nitric oxide synthase and then exposed to two high-salt (HS) challenges, separated by a wash-out period (l-NAME-HS1-HS2 protocol). Pretreatment with l-NAME induced salt-sensitive hypertension in naïve mice. Blood pressure increased during the HS challenges and returned to baseline in the intermittent washout period. Administration of FTY720 during the second HS challenge blunted the second hypertensive response. The group theorized that this protection from hypertension was due to decreased T cell migration to the kidneys. They demonstrated that following FTY720 treatment, CD8^+^ effector memory T cells accumulated in the bone marrow, supporting the concept that the drug blocks their egression to the periphery. Finally, the group used elegant T cell adoptive transfer studies to examine whether memory T cells could program salt-sensitive hypertension. Naïve mice, which received memory T cells from mice exposed to the l-NAME-HS1-HS2 protocol, developed hypertension when fed a HS diet. In contrast, the transfer of effector memory T cells from mice treated with FTY720 during the second HS challenge did not result in salt-sensitive hypertension. This suggests that memory T cells can induce salt sensitivity following exposure to hypertension (Itani et al., 2016a; Itani et al., 2016b). These data add to a growing body of research that highlights the importance of chemokine signaling in hypertension (Liao et al., 2008; Mikolajczyk et al., 2016; Rudemiller and Crowley, 2017) and suggests that blocking memory T cell egression may be a therapeutic approach to protect the kidney from hypertensive injury.

## Targeting renal inflammation for the treatment of hypertension

The review by Chaudhari et al. provides a comprehensive overview of how targeting renal inflammation may have therapeutic potential for the treatment of hypertension. The authors begin by highlighting the anti-inflammatory properties of many first-line treatments for hypertension. They propose that reducing renal inflammation may be responsible for some of these medications’ antihypertensive effects. The review focuses next on the vast experimental evidence supporting the idea that kidney inflammation contributes to the development and maintenance of hypertension. Finally, the article discusses how a renal involvement in autoimmune conditions often leads to hypertension. Systemic lupus erythematosus (SLE) is an autoimmune condition affecting the kidneys (Lupus nephritis) in up to 50% of cases. When this occurs, it is frequently associated with the development of hypertension (Singh and Saxena, 2009; Cojocaru et al., 2011). Therefore, SLE is a model to study the link between renal inflammation and hypertension. Indeed, studies using female NZBWF1 mice, a murine model of SLE, have demonstrated the critical role of the immune system in hypertension associated with autoimmunity (Mathis et al., 2014; Mathis et al., 2017; Taylor and Ryan, 2017; Taylor et al., 2019).

## The effect of dietary shifts on hypertension and cardiovascular disease

The review by Labban et al. explores the roles that high sodium and fructose diets play in the development of hypertension and cardiovascular disease. They focus on Lebanon, where there has been a three-fold increase in the incidence of hypertension in the past decade. The authors draw a connection between this increase and the increased consumption of processed foods high in sodium and fructose. Next, the article summarizes the complex mechanisms underlying sodium- and fructose-induced hypertension. Both high-sodium and high-fructose intake affect the immune system, and renal inflammation is a characteristic of experimental hypertension induced by high-sodium and high-fructose diets.

Interestingly, innate immune cells scan the gut epithelia for antigens and are abundant in the intestinal mucosa (Pott and Hornef, 2012). Recent studies found that consuming a high-salt diet reduced the abundance of *Lactobacillus* in the gut microbiome. In humans, this was associated with increased blood pressure and increased circulating T_H_17 cells (Wilck et al., 2017). Therefore, the interindividual heterogeneity in blood pressure responses to salt may be influenced by changes in the microbiome. The review by Labban et al. concludes by discussing strategies to reduce sodium and fructose consumption in Lebanon. The authors address challenges, including the lack of both existing initiatives and national targets for consumption levels. They advocate for a multifaceted approach targeting the food industry, increasing consumer awareness, and improving food labels. It is anticipated that reducing dietary fructose and sodium will lessen the burden of hypertension and cardiovascular disease.

## Conclusion

The articles in the Research Topic contribute to a growing body of evidence implicating the immune system in the development of hypertensive disorders. What is apparent from the articles is the complex nature of hypertension, which is driven by a range of factors, including, in some incidences, inflammation. Therefore, identifying biomarkers that enable the stratification of patients into subpopulations: those in which blood pressure is reduced by anti-inflammatory therapies *versus* those in which it is not, is a valuable area of future research.

## References

[B1] Banek C. T. Gauthier M. M. Van Helden D. A. Fink G. D. Osborn J. W. (2019). Renal inflammation in DOCA-salt hypertension. Hypertension 73, 1079–1086. 10.1161/HYPERTENSIONAHA.119.12762 30879356 PMC6540804

[B2] Boesen E. I. Williams D. L. Pollock J. S. Pollock D. M. (2010). Immunosuppression with mycophenolate mofetil attenuates the development of hypertension and albuminuria in deoxycorticosterone acetate-salt hypertensive rats. Clin. Exp. Pharmacol. Physiol. 37, 1016–1022. 10.1111/j.1440-1681.2010.05428.x 20626757 PMC3612979

[B3] Cojocaru M. Cojocaru I. M. Silosi I. Vrabie C. D. (2011). Manifestations of systemic lupus erythematosus. Maedica (Bucur) 6, 330–336. 22879850 PMC3391953

[B4] Crowley S. D. Song Y. S. Lin E. E. Griffiths R. Kim H. S. Ruiz P. (2010). Lymphocyte responses exacerbate angiotensin II-dependent hypertension. Am. J. Physiol. Regul. Integr. Comp. Physiol. 298, R1089–R1097. 10.1152/ajpregu.00373.2009 20147609 PMC4422347

[B5] De Miguel C. Guo C. Lund H. Feng D. Mattson D. L. (2011). Infiltrating T lymphocytes in the kidney increase oxidative stress and participate in the development of hypertension and renal disease. Am. J. Physiol. Ren. Physiol. 300, F734–F742. 10.1152/ajprenal.00454.2010 PMC3064138 21159736

[B6] Ertuglu L. A. Kirabo A. (2022). Dendritic cell epithelial sodium channel in inflammation, salt-sensitive hypertension, and kidney damage. Kidney 3, 1620–1629. 10.34067/KID.0001272022 PMC9528365 36245645

[B7] Evans L. C. Petrova G. Kurth T. Yang C. Bukowy J. D. Mattson D. L. (2017). Increased perfusion pressure drives renal T-cell infiltration in the dahl salt-sensitive rat. Hypertension 70, 543–551. 10.1161/HYPERTENSIONAHA.117.09208 28696224 PMC5589123

[B8] Garris C. S. Blaho V. A. Hla T. Han M. H. (2014). Sphingosine-1-phosphate receptor 1 signalling in T cells: Trafficking and beyond. Immunology 142, 347–353. 10.1111/imm.12272 24597601 PMC4080950

[B9] Guzik T. J. Hoch N. E. Brown K. A. Mccann L. A. Rahman A. Dikalov S. (2007). Role of the T cell in the Genesis of angiotensin II induced hypertension and vascular dysfunction. J. Exp. Med. 204, 2449–2460. 10.1084/jem.20070657 17875676 PMC2118469

[B10] Harrison D. G. Guzik T. J. Lob H. E. Madhur M. S. Marvar P. J. Thabet S. R. (2011). Inflammation, immunity, and hypertension. Hypertension 57, 132–140. 10.1161/HYPERTENSIONAHA.110.163576 21149826 PMC3028593

[B11] Hughson M. D. Gobe G. C. Hoy W. E. Manning R. D. Douglas-Denton R. Bertram J. F. (2008). Associations of glomerular number and birth weight with clinicopathological features of African Americans and whites. Am. J. Kidney Dis. 52, 18–28. 10.1053/j.ajkd.2008.03.023 18514988

[B12] Itani H. A. Mcmaster W. G., JR. Saleh M. A. Nazarewicz R. R. Mikolajczyk T. P. Kaszuba A. M. (2016a). Activation of human T cells in hypertension: Studies of humanized mice and hypertensive humans. Hypertension 68, 123–132. 10.1161/HYPERTENSIONAHA.116.07237 27217403 PMC4900908

[B13] Itani H. A. Xiao L. Saleh M. A. Wu J. Pilkinton M. A. Dale B. L. (2016b). CD70 exacerbates blood pressure elevation and renal damage in response to repeated hypertensive stimuli. Circ. Res. 118, 1233–1243. 10.1161/CIRCRESAHA.115.308111 26988069 PMC4833561

[B14] Itani M. M. Jarrah H. Maaliki D. Radwan Z. Farhat R. Itani H. A. (2022). Sphingosine 1 phosphate promotes hypertension specific memory T cell trafficking in response to repeated hypertensive challenges. Front. Physiol. 13, 930487. 10.3389/fphys.2022.930487 36160839 PMC9490048

[B15] Kearney P. M. Whelton M. Reynolds K. Muntner P. Whelton P. K. He J. (2005). Global burden of hypertension: Analysis of worldwide data. Lancet 365, 217–223. 10.1016/S0140-6736(05)17741-1 15652604

[B16] Liao T. D. Yang X. P. Liu Y. H. Shesely E. G. Cavasin M. A. Kuziel W. A. (2008). Role of inflammation in the development of renal damage and dysfunction in angiotensin II-induced hypertension. Hypertension 52, 256–263. 10.1161/HYPERTENSIONAHA.108.112706 18541733 PMC2562771

[B17] Maaliki D. Itani M. M. Itani H. A. (2022). Pathophysiology and genetics of salt-sensitive hypertension. Front. Physiol. 13, 1001434. 10.3389/fphys.2022.1001434 36176775 PMC9513236

[B18] Madhur M. S. Elijovich F. Alexander M. R. Pitzer A. Ishimwe J. Van Beusecum J. P. (2021). Hypertension: Do inflammation and immunity hold the key to solving this epidemic? Circ. Res. 128, 908–933. 10.1161/CIRCRESAHA.121.318052 33793336 PMC8023750

[B19] Madhur M. S. Lob H. E. Mccann L. A. Iwakura Y. Blinder Y. Guzik T. J. (2010). Interleukin 17 promotes angiotensin II-induced hypertension and vascular dysfunction. Hypertension 55, 500–507. 10.1161/HYPERTENSIONAHA.109.145094 20038749 PMC2819301

[B20] Mathis K. W. Taylor E. B. Ryan M. J. (2017). Anti-CD3 antibody therapy attenuates the progression of hypertension in female mice with systemic lupus erythematosus. Pharmacol. Res. 120, 252–257. 10.1016/j.phrs.2017.04.005 28400152 PMC5836511

[B21] Mathis K. W. Wallace K. Flynn E. R. Maric-Bilkan C. Lamarca B. Ryan M. J. (2014). Preventing autoimmunity protects against the development of hypertension and renal injury. Hypertension 64, 792–800. 10.1161/HYPERTENSIONAHA.114.04006 25024282 PMC4353646

[B22] Matloubian M. Lo C. G. Cinamon G. Lesneski M. J. Xu Y. Brinkmann V. (2004). Lymphocyte egress from thymus and peripheral lymphoid organs is dependent on S1P receptor 1. Nature 427, 355–360. 10.1038/nature02284 14737169

[B23] Mattson D. L. (2014). Infiltrating immune cells in the kidney in salt-sensitive hypertension and renal injury. Am. J. Physiol. Ren. Physiol. 307, F499–F508. 10.1152/ajprenal.00258.2014 PMC4154114 25007871

[B24] Mattson D. L. Lund H. Guo C. Rudemiller N. Geurts A. M. Jacob H. (2013). Genetic mutation of recombination activating gene 1 in Dahl salt-sensitive rats attenuates hypertension and renal damage. Am. J. Physiol. Regul. Integr. Comp. Physiol. 304, R407–R414. 10.1152/ajpregu.00304.2012 23364523 PMC3602820

[B25] Mikolajczyk T. P. Nosalski R. Szczepaniak P. Budzyn K. Osmenda G. Skiba D. (2016). Role of chemokine RANTES in the regulation of perivascular inflammation, T-cell accumulation, and vascular dysfunction in hypertension. FASEB J. 30, 1987–1999. 10.1096/fj.201500088R 26873938 PMC4836375

[B26] Moes A. D. Severs D. Verdonk K. Der-Lubbe N. V. Zietse R. Danser A. H. J. (2018). Mycophenolate mofetil attenuates DOCA-salt hypertension: Effects on vascular tone. Front. Physiol. 9, 578. 10.3389/fphys.2018.00578 29867591 PMC5968119

[B27] Pott J. Hornef M. (2012). Innate immune signalling at the intestinal epithelium in homeostasis and disease. EMBO Rep. 13, 684–698. 10.1038/embor.2012.96 22801555 PMC3410395

[B28] Rudemiller N. P. Crowley S. D. (2017). The role of chemokines in hypertension and consequent target organ damage. Pharmacol. Res. 119, 404–411. 10.1016/j.phrs.2017.02.026 28279813 PMC5424532

[B29] Schutte A. E. Srinivasapura Venkateshmurthy N. Mohan S. Prabhakaran D. (2021). Hypertension in low- and middle-income countries. Circ. Res. 128, 808–826. 10.1161/CIRCRESAHA.120.318729 33793340 PMC8091106

[B30] Singh S. Saxena R. (2009). Lupus nephritis. Am. J. Med. Sci. 337, 451–460. 10.1097/MAJ.0b013e3181907b3d 19390431

[B31] Taylor E. B. Ryan M. J. (2017). Immunosuppression with mycophenolate mofetil attenuates hypertension in an experimental model of autoimmune disease. J. Am. Heart Assoc. 6, e005394. 10.1161/JAHA.116.005394 28242635 PMC5524041

[B32] Taylor E. B. Sasser J. M. Maeda K. J. Ryan M. J. (2019). Expansion of regulatory T cells using low-dose interleukin-2 attenuates hypertension in an experimental model of systemic lupus erythematosus. Am. J. Physiol. Ren. Physiol. 317, F1274–F1284. 10.1152/ajprenal.00616.2018 PMC6879936 30892934

[B33] Wilck N. Matus M. G. Kearney S. M. Olesen S. W. Forslund K. Bartolomaeus H. (2017). Salt-responsive gut commensal modulates TH17 axis and disease. Nature 551, 585–589. 10.1038/nature24628 29143823 PMC6070150

[B34] Xiao L. Kirabo A. Wu J. Saleh M. A. Zhu L. Wang F. (2015). Renal denervation prevents immune cell activation and renal inflammation in angiotensin II-induced hypertension. Circ. Res. 117, 547–557. 10.1161/CIRCRESAHA.115.306010 26156232 PMC4629828

